# Seizure and Pulmonary Embolism: A Differential That Can Save a Life

**DOI:** 10.1155/2017/3408795

**Published:** 2017-12-17

**Authors:** Seyedmohammad Pourshahid, Sneha Dedhia, Shakeeb Hakim, Mohamed Barakat, Dennis Genin

**Affiliations:** Department of Medicine, Icahn School of Medicine at Mount Sinai/NYC Health + Hospitals/Queens, New York, NY, USA

## Abstract

Seizures is a relatively common presentation with a wide differential diagnosis. However, seizures presenting secondary to underlying pulmonary emboli are rare and, without prompt recognition and management, this easily treatable condition can be potentially fatal. The few available case reports discussing seizures and PE reveal a high mortality rate which underscores the importance of prompt diagnosis. A 38-year-old woman presented to the emergency room having experienced loss of consciousness and a generalized tonic-clonic seizure at home. In the emergency room, her presenting signs and symptoms included tachycardia, worsening dyspnea, mild hypoxemia, and elevated D-dimer. Pertinent history findings revealed she recently received depot hormonal contraceptive treatments. Her initial workup included an EKG which showed sinus tachycardia without evidence of right heart strain. Subsequently a chest CT with angiography revealed massive bilateral pulmonary emboli. DVT studies also revealed a unilateral acute DVT. The patient was promptly started on therapeutic anticoagulation and stabilized. Fortunately, the patient remained symptom-free and eventually was discharged with close follow-up. The goal of this report is to make clinicians more aware of the possibility that seizures, along with the appropriate clinical findings, can be caused by acute PE.

## 1. Introduction

Seizures is a relatively common presentation with a wide differential diagnosis. However, seizures presenting secondary to underlying pulmonary embolism (PE) are rare and, without prompt recognition and management, this easily treatable condition can be potentially fatal. PE is associated with approximately 1.3% of deaths in the United States. Without treatment the mortality rate increases up to 30% [1]. The clinical presentation of pulmonary embolism (PE) can be varied, making the diagnosis often underrecognized. In this article, a case of new onset seizure is discussed whereby a diagnosis of PE as the underlying cause was successfully managed. Also seven previous case reports were reviewed and the associated mortality rate was calculated.

## 2. Case Report

A 38-year-old African American female with past medical history of morbid obesity and epilepsy, on Depomedroxy progesterone injections for birth control, was brought to the ED along with her husband for an episode of loss of consciousness associated with abnormal body movements. She was found by her husband on bathroom floor having generalized body movements with urinary incontinence. She regained full consciousness within 10 minutes after a brief period of confusion. She was reporting shortness of breath and the sensation of palpitations after the episode of seizure-like activity. She had reported about 1 month prior to presentation having a seizure episode but had not resumed antiepileptic medications. She only used antiepileptic medications as a child and stopped taking them as an adult.

Physical exam was significant for sinus tachycardia and a room air saturation of 94%. No focal neurological deficit was noted. The patient was not complaining of shortness of breath at the initial presentation in the ED. Given the patient's risk factors, for example, hormonal contraceptive use, sedentary lifestyle, and relative hypoxemia on room air, the patient was sent for CTA which revealed massive PE with bilateral pulmonary emboli. Patient underwent lower extremity venous duplex showing acute DVT of the right popliteal vein. EEG was performed while inpatient was negative for epileptiform activity. She was discharged in stable condition on 7th day of admission on therapeutic lovenox injection and antiepileptic medications. She has continued to do well and remains seizure-free on follow-up visits.

## 3. Discussion

Seizures are reported in the literature as a presenting symptom of various cardiopulmonary pathologies such as long QT syndrome [2], severe bradycardia [3], systemic hypertension [4], and pulmonary embolism [5]. Acute PE presenting with new onset seizure occurs in less than 1 percent of cases [5].

The current theory for the pathophysiology was developed by Marine and Goldhaber in 1997 explaining that seizure was secondary to hypoxia, metabolic and respiratory acidosis in the setting of right heart, and respiratory failure and transient cerebral hypoperfusion similar to a cardiogenic seizure mechanism. The majority of literature discussing PE presenting as seizures is found in case reports. Review of previous case reports including Fred and Yang in 1995 [5], Marine and Goldhaber in 1997 [6], Meyer in 2009 [7], Shah and Darwent in 2009 [8], Volz and Jasani in 2014 [9], and Ching et al. in 2015 [10] revealed 37.5% (3 out of 8) of the cases did not survive the hospital encounter, which is consistent with both massive PE mortality rates of 25–65% [11] and untreated PE mortality rate of 30% [1]. The mortality rate for submassive and low risk PE is 3% and 1%, respectively. Out of the eight reviewed cases, 6 were not considered massive PE (either submassive or low risk). For the other two cases, clinical findings reported were not sufficient to accurately classify the PE. Although given small sample sizes of the case reports, it is hard to ascertain any statistical significance; however, review of PE case presenting with seizure is significantly higher than submassive and low risk PE groups and at the same time the description of most of reported cases does not qualify for massive PE. Delay in the treatment of PE increases the mortality up to 30% for untreated PE. Her symptoms of hypoxia, tachycardia, and shortness of breath can be attributed to a postictal phase or even obesity hypoventilation syndrome, which would have delayed the diagnosis and hence the treatment, if not for careful history taking and maintaining a wide differential diagnosis.

In conclusion, PE is often a diagnosis of exclusion, but as clinician we must be ever vigilant not to ignore the obvious and when confronted with a clinical scenario suggestive of PE it warrants detailed evaluation.
